# On Defect Evolution in EBM Additively Manufactured Ti-6Al-4V via In Situ Investigations

**DOI:** 10.3390/ma17122888

**Published:** 2024-06-13

**Authors:** Wei Sun, Ming Li, Hezong Li

**Affiliations:** 1 School of Intelligent Manufacturing and Electronic Engineering, Wenzhou University of Technology, Wenzhou 325035, China; 2 State Key Laboratory of Clean and Efficient Turbomachinery Power Equipment, Northwestern Polytechnical University, Xi’an 710072, China; 3 School of Mechanical and Equipment Engineering, Hebei University of Engineering, Handan 056038, China

**Keywords:** additive manufacturing, EBM-Ti-6Al-4V, defect, damage mechanism, in situ

## Abstract

This study concerned the in situ investigation of the defect evolution and fracture mechanism of additively manufactured (AM) Ti-6Al-4V under uniaxial tensile tests. In order to achieve this, microstructure characterization was initially carried out in order to identify the defects within the matrix of the candidate material. In situ testing was then performed, focusing on the spherical defect to observe its evolution under tensile loading. It was found that, before the fracture stage, the geometric evolution of the spherical defect towards an ellipse shape was dominated by the load in the tensile direction. In addition, the slip band density was found to be aggravated near the spherical defect due to the geometric discontinuity-induced stress concentration. During the fracture process, the defect geometry evolved as an irregular shape, which was mainly attributed to the micro-void-induced localized multi-axial stress state. The fracture analysis indicated that defects play a key role in crack initiation, leading to the fracture of LPBF materials.

## 1. Introduction

The dual-phase (α/β), lightweight Ti-6Al-4V alloy has excellent comprehensive physical and mechanical properties, such as high strength retention, high fracture toughness, and excellent oxidation resistance at high temperatures [1,2,3,4], making it the preferred material for use in some specific environments—for example, in some common applications that require a low density and excellent corrosion resistance, such as the aerospace industry and biomechanical applications (human implants and prostheses). In the aerospace industry, the geometric design of most parts has a complex structural form, and it is well known that the high-temperature deformation ability of Ti-6Al-4V alloy is very poor, which makes it difficult to manufacture this type of alloy using traditional forging methods. Therefore, in order to manufacture complex structural parts under an increasing demand, researchers have adopted the recently emerging AM technology to manufacture such alloys [5,6]. Laser-based powder bed fusion (LBPF) is an emerging near-net-shape AM technology that can produce metal parts with complex geometric shapes and high densities at a fast speed, opening up new paths for technological breakthroughs in the aviation industry [7,8]. The basic principle of LBPF technology is to use a laser to melt a metal powder and then deposit it layer by layer. Compared with traditional manufacturing methods, layered manufacturing technology provides many other potential advantages, such as high material utilization efficiency, more complex geometric structures, and a reduction in unnecessary subsequent cutting steps.

**Figure 1:**
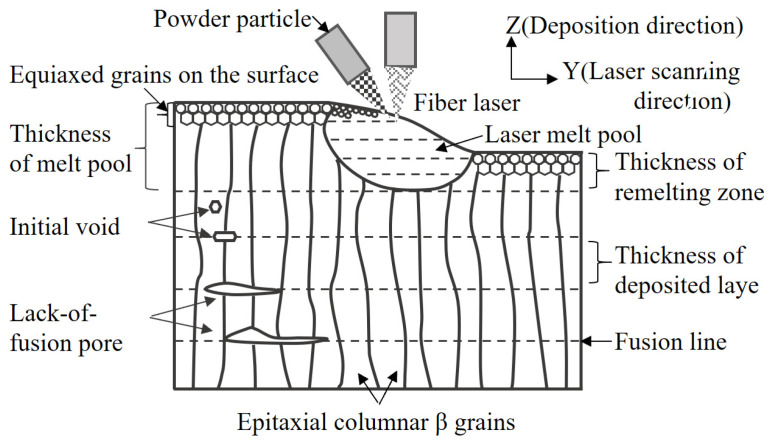
A schematic diagram of defect generation during the LBPF process.

In the LBPF process, compared to the corresponding traditional forging materials, the cooling rate is very fast, leading to the formation of pores or defects. Defects in AM materials are difficult to avoid and may be caused by many factors, such as keyholes, trapped gas pores, and a lack of fusion (LoF). Figure 1 shows a schematic diagram of the generation of defects during the LBPF process. Haynes [9,10] found, through experiments, that defects have a significant impact on the tensile strength and ductility. In addition, due to defects [11,12], the fatigue life can also be significantly affected. For relatively brittle materials, it is suggested that trace defects are particularly detrimental to their ductility [10]. Meanwhile, other works have presented contrasting views. For example, in the work performed by Osetsky [13], it was found that defects may have an enhancing effect [13], even enhancing the tensile ductility due to dislocation–void interactions [14]. As is well known, the selection of the laser parameters during LBPF processing, such as the power and speed, layer thickness, scanning strategy, and support structure, not only affects the grain size [15] but also determines the defect content [16]. Due to the widespread presence of defects, even in high-density samples, they will play a crucial role in determining the mechanical and fracture behavior of AM samples throughout their entire service life. Therefore, the defect characteristics in AM materials and their geometric evolution and fracture mechanisms under mechanical loads are worthy of in-depth research.

The aim of this study is to identify the degree and mechanism of influence of defects regarding the mechanical properties and failure behavior of LBPF materials during room-temperature tensile deformation; evaluate the potential risks of defects on the material’s performance; and reveal the microstructural evolution phenomenon and crack initiation and propagation processes of LBPF materials during room-temperature tensile deformation.

## 2. Materials and Experimental Methods

### 2.1. Materials

Ti-6Al-4V alloy has a dual-phase structure with a hexagonal closely packed (HCP) α-phase structure and a body-centered cubic structure (BCC) β-phase. In this study, Ti-6Al-4V (grade 23) ultra-low interlayer (ELI) plasma atomized spherical powder, provided by LPW Technology, was used for the additive manufacturing of stretched samples. The particle size of the powder was distributed between 15 μm and 45 μm, with an average of 30 μm. The elemental composition of the raw materials [17] used in this study met the requirements of ASTM B348 (weight%), as shown in Table 1.

The in situ SEM tensile specimens used in this experiment were prepared using LBPF technology. Based on the computer-aided design model of the parts to be processed, a high-energy laser beam was used to scan and melt the selected area of the alloy powder, and a thin layer consisting of a cross-section of the part in a certain shape was deposited. It was stacked layer by layer until a high-density three-dimensional part was produced, which was constructed using a commercial EOS M280 a.m. machine. The inner cavity size of the EOS machine was 250 × 250 × 325 mm^3^, and the construction process was run under argon protection. The substrate was selected as Grade 5 Ti-6A-4V, and it was preheated to 40 °C before the experiment. The construction process of this machine uses an IPG fiber laser source, and its maximum power can reach 10,000 W. During the construction process, the conveying of the powder and the transfer of heat energy from the light source are both based on the principle of high-precision coaxial powder feeding. Through this working principle, it is easy to control the synchronous deflection of the beam and raw materials. The output powder and light source energy supply occur simultaneously in the required position of the molten pool, layer by layer, on the constructed part. Prior to the start of the experiment, all process steps were pre-set to obtain high-quality dense bulk samples with a uniform deposition layer and as few pores as possible. After processing, the supporting substrate was separated and sandblasted with water to remove loosely bonded powder on the sample surface and improve its surface roughness. Afterwards, stress relief annealing was carried out for 3 h in argon gas protection above 630 °C, and then the furnace was cooled to room temperature. The construction parameters of this sample are shown in Table 2.

### 2.2. Sample Preparation

**Figure 2:**
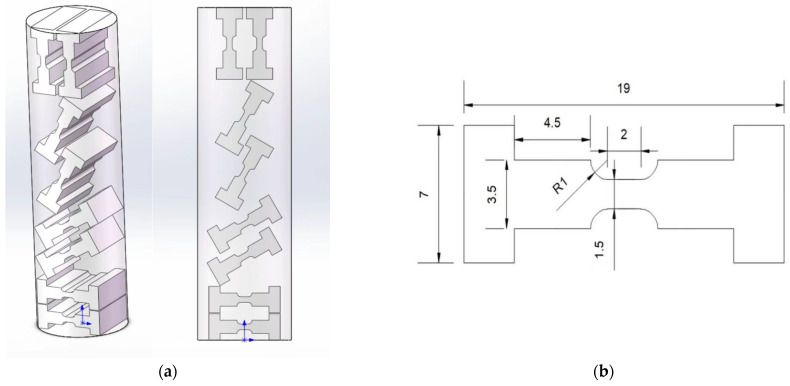
(**a**) A schematic diagram of in situ specimen extraction; (**b**) the specimen dimensions with a thickness of 0.5 mm (all marked dimensions are in mm). The arrows in (**a**) represent the X-axis (horizontal) and Y-axis (vertical) in the Cartesian coordinate system.

In this work, an in situ SEM specimen was cut from the LPBF substrate using the electric discharge wire cutting method. Figure 2a shows a schematic diagram to illustrate the in situ dog-bone specimen extraction. It should be noted that, in this work, we only focus on the in situ behaviour of the materials in the horizontal direction, without considering the orientation effect. Figure 2b shows the geometry and dimensions of the dog-bone specimen used for the in situ test. As can be seen, the parallel gauge length was about 2.0 mm, with a thickness of 0.5 mm and a gauge width of 1.5 mm. The remaining dimensions are given in Figure 2b. The load–displacement response can be measured at the ends of the fillets. For metallographic characterization, the sample sections were first polished with SiC papers (p360, 600, 1000, and 1500 particle sizes); they were then finely polished with a plane cloth (5 mm) and an alpha cloth (1.5 mm) in a diamond suspension and finally finely polished with a chemical cloth in a colloidal SiO_2_ solution, to facilitate the preliminary microscopic examination of the microstructure.

### 2.3. Experimental Set-Up

**Figure 3:**
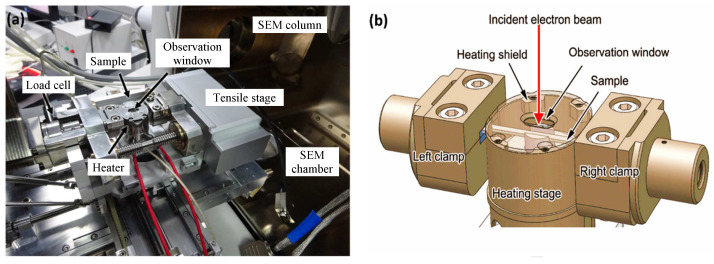
(**a**) An illustration to show the in situ SEM test setup; (**b**) the detailed structure of the stretching stage.

Before tensile testing, a ZEISS Gemini SEM300 scanning electron microscope (SEM) from Oxford Symmetry was utilized for microscopy characterization. The SEM working parameters included a working distance of 10 mm, voltage of 20 kV, and probe current of 15 nA, which were used for image capture (including the parameters during in situ SEM testing). After the in situ tensile test, the surface morphology of the sample after fracture was observed and photographed on the same SEM. The sample was placed on a specific microtensile test bench in the SEM chamber to observe its in situ deformation and microstructure changes under a uniaxial tensile load at room temperature, thus directly studying its microstructure evolution and deformation process. The polished dog-bone specimen was clamped at both ends by specifically designed and manufactured clamps, as shown in Figure 3a. As mentioned above, the entire stretching process was carried out on a specific microtensile test bench in the SEM chamber until the final fracture of the specimen. During the stretching process, the displacement rate was fixed at 1 µm/s and the temperature was maintained at room temperature throughout the entire experiment. The detailed structure of the stretching stage is shown in the schematic in Figure 3b.

## 3. Results

### 3.1. Defect Characterization of As-Received Ti-6Al-4V

**Figure 4:**
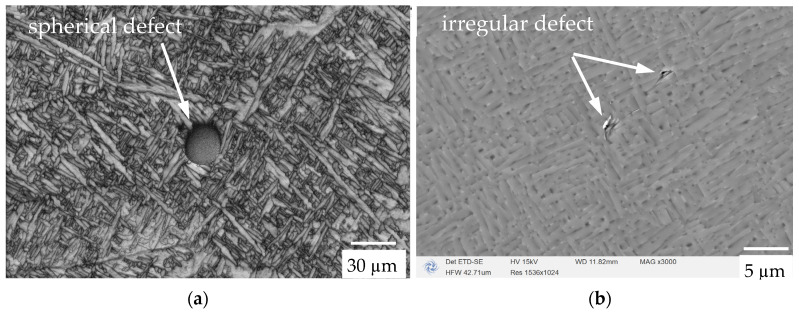
SEM images obtained from the in situ dog-bone sample to show the defect morphology in LPBF Ti-6Al-4V: (**a**) spherical defect and (**b**) irregular defect.

The SEM micrograph of the sample surface parallel to the in situ tensile direction (perpendicular to the construction direction) is shown in Figure 4. As can be seen, the main characteristic of the microstructure is that it is generated based on the prior columnar β grains, with an average width between 80 and 100 µm, and its form consists of the epitaxial growth of the prior β grain boundaries across multiple layers. From the SEM micrograph, an acicular martensitic α’ structure (needle-like) can also be seen. In the cross-section perpendicular to the construction direction, these α’ needles appear to cross the boundaries of the prior β grains. Due to the fact that the prior β grains grow along the deposition direction, this prior β grain growth mode will lead to an inhomogeneous microstructure and correspondingly affect the mechanical properties. The defects observed from the SEM micrographs are spherical and irregularly shaped, as shown in Figure 4a,b. Researchers have conducted X-ray CT scans on this additive manufacturing material in the past [18] and found that the volume content of the defects in the representative, vertically constructed samples was 0.034% (vol). Most defects in horizontally constructed samples are small and spherical (pores). The irregularly shaped defect was not focused on in the in situ work.

Through the metallographic images and SEM observations, it can be seen that due to the advantages of LBPF technology, the actual content of the pore defects in the samples used in this experiment was much lower than expected, regardless of whether they were spherical or irregular defects. Therefore, during the room-temperature tensile deformation and fracture process of the sample, the defects did not have a significant impact on its mechanical properties. This was validated in the observation of the in situ tensile results later in this study.

### 3.2. In Situ Observation and Analysis

#### 3.2.1. Uniaxial Tensile Results

**Figure 5:**
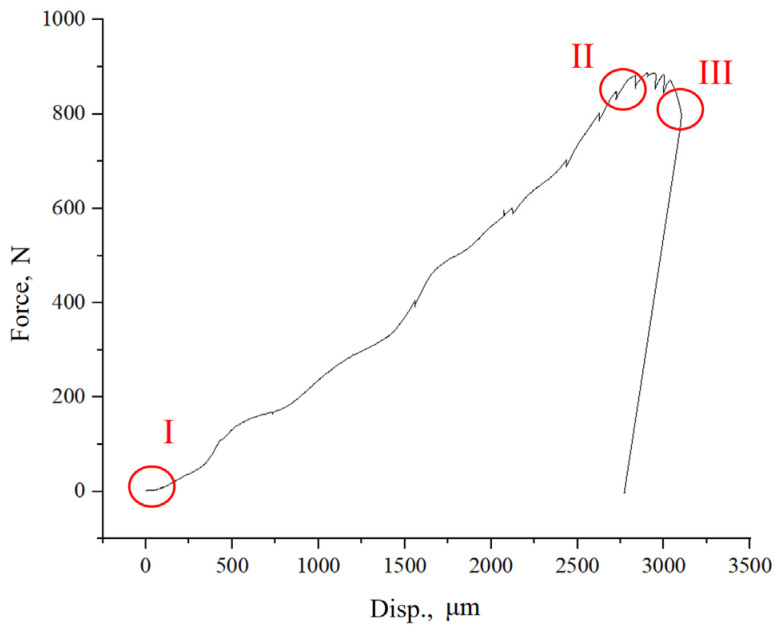
Tensile response in the in situ experiment in which imaging was performed at the stress levels marked on the curve (I, II and III).

In situ SEM tensile testing is one of the best methods to decipher the relationship between a material’s mechanical properties, its microstructure, and its defect evolution, as it allows for the real-time synchronous observation of the evolution of the internal microstructure, changes in the original defects, and the origin and growth of new defects during the deformation process of the specimen caused by the gradual increase in the external load. The in situ samples in this work were stretched at a deformation rate of 1 μm/s at room temperature. During the stretching process, at some displacement points, shown in Figure 5, we paused the loading of the specimen and maintained the load, and we continued the loading process after completing SEM image acquisition. From the tensile curve, it can be seen that in the early stage of stretching, until the elongation of the sample reached about 2600 μm, the applied tensile force of the sample increased linearly with its elongation. When the elongation of the sample reached about 2600 μm, yielding was observed on the tensile curve, and the magnitude of the applied tensile force was about 850 N. Within a small range thereafter, necking occurred in the tensile specimen, followed by a fracture at a position with elongation of approximately 3000 μm and an applied tensile force of approximately 800 N.

During the whole in situ process, three typical time points, as illustrated in Figure 5 with the symbols of I, II, and III, were selected in order to examine the spherical defect’s evolution. These were located in the original stage without loading, the moment before fracture after large deformation, and the moment after fracture. The microstructure deformation characteristics in the samples corresponding to these three time points accurately reflect the internal structural changes of the samples under external forces, as well as the process of crack generation and propagation. After completing SEM image acquisition, the stretching process was continued until the specimen fractured. Severe specimen deformation was observed during the in situ loading stage of this work.

#### 3.2.2. In Situ Characterization of Defect Evolution

**Figure 6:**
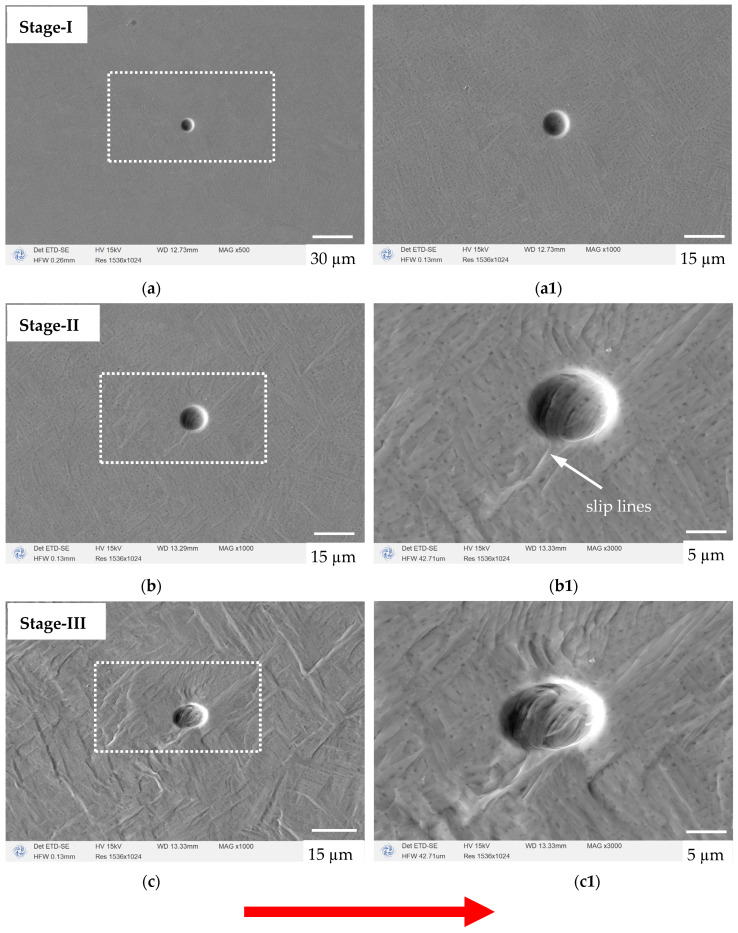
SEM observations focusing on the spherical defect’s evolution; the left is an overview of the defect and the right is a magnified view. The loading direction is shown by the red arrow. (**a**,**a1**) are images of stage-I in Figure 5, where the applied force is 0; (**b**,**b1**) are images of stage-II in Figure 5, where the applied force is 880 N; (**c**,**c1**) are images of stage-III in Figure 5, where the applied force is 800 N.

The microstructure’s evolution before and after straining, corresponding to Figure 5, is shown in Figure 6, where Figure 6a1,b1,c1 are enlarged images of the corresponding white dashed boxes in Figure 6a–c. Noted that the loading occurred along the horizontal direction, as indicated by the red arrow. As can be seen, the applied force acting on the tensile specimen increased from 0 to 850 N, and no significant plastic deformation was observed. For example, the polished surface of the sample was smooth before loading, and no traces of plastic deformation were observed; see Figure 6a,a1 (stage I). This indicates that the specimen was in an elastic deformation state when the load was less than 850 N.

When the applied stress reached 880 N, it can be seen from Figure 6b,b1 (stage II) that significant plastic deformation occurred inside the specimen, leading to some grains’ distortion. Since the microstructure was composed of prior β columnar grains and an α’ phase existing in the form of clusters, it could be found that the grooves formed by the slip lines undergoing plastic deformation were mainly distributed at the edges of the prior β columnar grains, the edges of the α’ clusters, and the boundaries of the needle-like α’ phase (see Figure 6b1).

As the external force increased, the plastic deformation continued to increase until the fracture of the sample. At this stage, severe twisting deformation occurred inside the grains, and the deformation area increased, as shown in Figure 6c,c1 (stage III). The plastic deformation caused significant undulations in the observation plane of the specimen, with crisscrossing slip lines distributed, which brought uneven grooves and raised platforms to the originally smooth plane of the specimen. Meanwhile, local shear deformation bands appeared at an angle of approximately 45° to the stretching direction (Figure 6c,c1).

It can be concluded here that, in areas where significant deformation occurred, the colonies were separated by slip lines, as shown in stage II and stage III. Moreover, the density of the slip lines was directly proportional to the magnitude of the strain, oriented parallel or perpendicular to each other. The slip lines were uniformly and dispersedly distributed in the matrix, and their coordinated sliding bore the overall elongation deformation of the specimen under the external forces. This could be confirmed by the fact that the initial spherical defect was eventually elongated into an ellipse shape during plastic deformation.

From the in situ experiment on the plastic deformation to fracture process of uniaxial tensile samples, it can be concluded that, in the early stage of deformation, almost all plastic deformation in the sample arose from the slip that occurred at the edges of the prior β columnar grains, the edges of the α’ clusters, and the boundaries of the needle-like α’ phase. At the same time, almost no plastic strain or plastic slip phenomenon was found inside the needle-like α’ phase as the matrix. This indicates that, at room temperature, the slip of dislocations within the needle-like α’ phase had higher activation energy than the slip of dislocations at the interface. These interfaces include the edges of the prior β columnar grains, the edges of the α’ clusters, and the boundaries of the needle-like α’ phase. The energy required to initiate the slip system at these interfaces is much lower than that inside the needle-like α’ phase. Moreover, from the fact that these three different boundaries are subjected to the overall elongation deformation of the specimen under external forces through their coordinated sliding, it can be concluded that the slip systems present in these three different boundaries have similar activation energies.

### 3.3. Fracture Fractography

**Figure 7:**
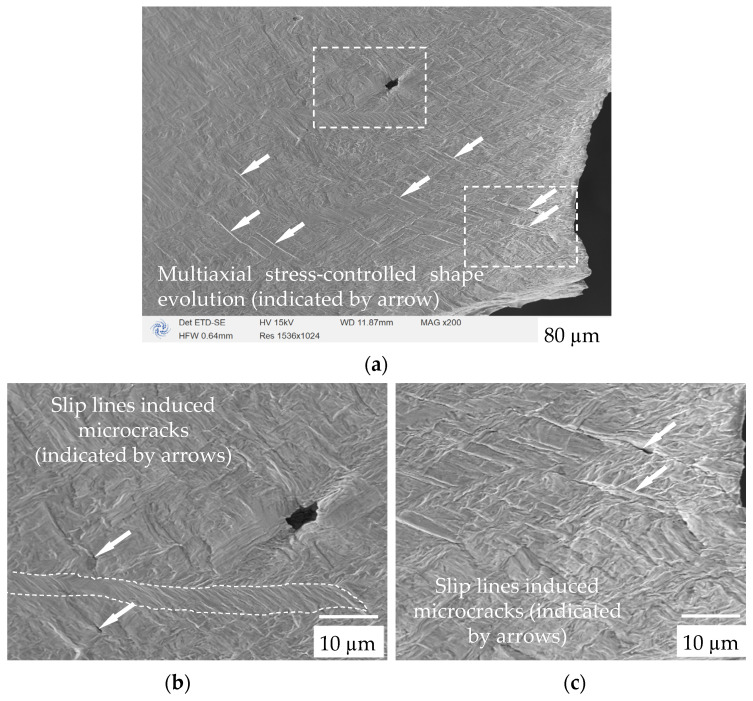
The defect and microstructure evolution at the fracture stage: (**a**) the focused spherical defect shape; (**b**) the microstructure morphology around the defect, showing the slip band-induced microcracks; and (**c**) the microcrack near the fracture surface. The loading direction is consistent with Figure 6.

Figure 7 shows the fractured surface of the dog-bone specimen. It can be seen in Figure 7a that the focused spherical defect changes significantly due to the microcrack-induced localized multi-axial stress state. Additionally, the microcracks caused by the localized slip lines present in the dog-bone sample are indicated by the white arrows. Compared to the horizontal direction (loading direction), it seems that all microcracks exhibit an approximately consistent angle distribution. Furthermore, the microcracks are mainly distributed at the edges of the initial β-shaped grains and the edges of the α’ cluster. The generation of such cracks is due to the strain-strengthening effect caused by the excessive dislocation slip exceeding the limit of dislocation accumulation that the corresponding slip system in the material can accommodate, resulting in the formation of crack nucleation [19]. Figure 7b,c are enlarged images corresponding to the dashed boxes in Figure 7a. It can be seen that the grains have been stretched into a long strip shape near the fracture surface, and the slip lines are distributed horizontally along the direction close to the stretching axis.

The initiation of these cracks indicates that, in the early stage of plastic deformation, the dislocation slip originating from the edges of the prior β columnar grains and the edges of the α’ clusters is not only the cause of the room-temperature tensile plastic deformation in the LBPF sample, but also the cause of the early crack initiation and propagation in the sample [20]. As the degree of plastic deformation increases, plastic strain and plastic slip phenomena, which are coordinated with the overall severe deformation of the sample, gradually begin to occur at the edges and interior of the needle-like α’ phase as the matrix. However, the magnitude of its deformation is not sufficient to cause the dislocation slip to reach the level of cracking. Observing the evolution of the microstructure during the room-temperature tensile process of the sample, it was found that as the deformation increased, the positions where the slip lines appeared inside the sample were distributed at the boundaries of these microsystems according to their volume. In other words, the slip lines were generated in the order of the edges of the prior β columnar grains, the edges of the α’ clusters, and the edges and interiors of the α’ phase. Figure 7c shows the area adjacent to the edge of the fracture, which has undergone severe plastic deformation, from which the twisted deformation of the needle-like α’ phase can be observed. The plastic slip within this smaller-scale microstructure (the needle-like α’ phase) changes the direction of the cracks generated by the slip at the edges of the previous two large-scale microstructures (with an angle of less than 45° in the tensile direction), thereby promoting the overall fracture of the specimen. Therefore, it can be concluded that the activation energy order of the slip systems located at different interfaces in the LBPF samples is as follows: the edges of the prior β columnar grains, the edges of the α’ clusters, and the boundaries and interiors of the needle-like α’ phase [21]. During the room-temperature tensile deformation process, these slip systems are sequentially activated, synergistically sliding and completing the plastic deformation and fracture of the sample.

**Figure 8:**
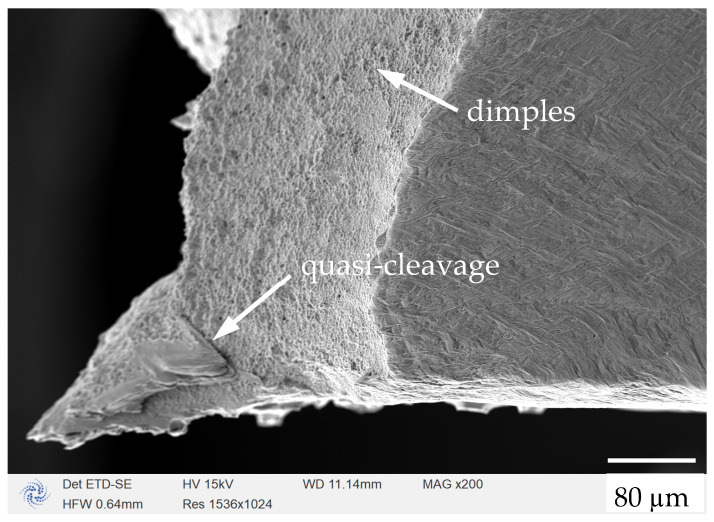
The fractured surface of the in situ dog-bone specimen.

The SEM image of the back-fractured surface of the specimen is shown in Figure 8. It can be seen the fracture surface exhibits a mixture of brittle and ductile fracture types, with a basically flat surface in the internal region (which is a brittle fracture feature) and some small shear slips towards the external region (which is a ductile fracture feature). Within the material, discrete regions caused by a lack of fusion and internal gas pores are common, which promote fracture during the stretching process. The microstructure photos also showed some quasi-cleavage fractures in the in situ tensile samples at room temperature, characterized by shallow and small-sized dimples. This indicates that the fracture mechanism of the material tested in this experiment was a mixed failure type of brittle fracture and ductile fracture. The slight flaking off of the metal was also observed on the fracture surface of the sample, which may have been due to the layer peeling phenomenon caused by the discontinuous non-fusion zone between the construction layers located at the core of the sample.

## 4. Conclusions

The tensile deformation process of AM Ti-6Al-4V alloy was observed using the in situ SEM method, with a focus on the evolution of spherical defects, the microstructure, and the slip lines in the LBPF samples during room-temperature tensile deformation. The plastic deformation mechanism and fracture process of the samples were analyzed. The following conclusions can be drawn.

- The geometric shape of the spherical defect was initially dominated by the loading in the tensile direction before the fracture stage, i.e., a transition from a spherical shape to an ellipse shape. Afterwards, an irregular shape was produced due to the microcrack-induced localized multi-axial stress state.
- The occurrence of defects is bound to produce localized stress concentration, leading to the aggravation of the localized slip lines, while the earliest microcrack formation is not relevant to the spherical defect. Early microcracks primarily initiated from the edges of the prior β columnar grains and the edges of the α’ clusters, and, as the deformation increased, microcracks were generated at the edges and interior of the needle-like α’ lamella.
- Due to the presence of colonies and grain boundaries in the plastic zone located at the tips of the microcracks, and their orientation perpendicular to the crack direction, the propagation of the microcracks was hindered.

## Figures and Tables

**Table 1:** Chemical composition of Ti-6Al-4V feedstock powder used in this study (in wt.%).

Al	V	C	O	N	H	Fe	Ti
6.40	4.0	0.02	0.12	0.02	0.002	0.19	Bal.

**Table 2:** Processing parameters selected for the fabrication of an LPBF in situ specimen.

Process Parameters	Values
Laser power (kW)	6
Laser thickness (mm)	0.7
Laser scan speed (mm/min)	800–1200
Hatch width (mm)	5
Hatch distance (mm)	1–3
Hatch overlap (mm)	0
Beam diameter (mm)	8
Powder feeding rate (g/h)	800–1000

## Data Availability

Data are contained within the article.

## References

[1] Vayssette B., Saintier N., Brugger C., El May M. (2020). Surface roughness effect of SLM and EBM Ti-6Al-4V on multiaxial high cycle fatigue. Theor. Appl. Fract. Mech..

[2] Qian G., Jian Z.M., Pan X.N., Berto F. (2020). In-situ investigation on fatigue behaviors of Ti-6Al-4V manufactured by selective laser melting. Int. J. Fatigue.

[3] Yánez A.M.F., Fiorucci Cuadrado A., Martel O., Monopoli D. (2020). Surface roughness effects on the fatigue behavior of gyroid cellular structures obtained by additive manufacturing. Int. J. Fatigue.

[4] Dinh T.D., Han S., Yaghoubi V., Xiang H., Erdelyi H., Craeghs T., Segers J., Paepegem W.W. (2021). Modeling detrimental effects of high surface roughness on the fatigue behavior of additively manufactured Ti-6Al-4V alloys. Int. J. Fatigue.

[5] Frazier W.E. (2014). Metal additive manufacturing: A review. J. Mater. Eng. Perform..

[6] Thompson S.M., Aspin Z.S., Shamsaei N., Elwany A., Bian L. (2015). Additive manufacturing of heat exchangers: A case study on a multi-layered Ti–6Al–4V oscillating heat pipe. Addit. Manuf..

[7] Li N., Huang S., Zhang G.D., Qin R.Y., Liu W., Xiong H.P., Shi G.Q., Blackburn J. (2019). Progress in additive manufacturing on new materials: A review. J. Mater. Sci. Technol..

[8] Bikas H., Stavropoulos P., Chryssolouris G. (2016). Additive manufacturing methods and modelling approaches: A critical review. Int. J. Adv. Manuf. Technol..

[9] Haynes R. (1971). Effect of porosity content on the tensile strength of porous materials. Powder Metall..

[10] Haynes R. (1991). Effects of porosity on the tensile strengths of sintered irons. Metal Powder Rep..

[11] Liu Y.J., Li S.J., Wang H.L., Hou W.T., Hao Y.L., Yang R., Sercombe T.B., Zhang L.C. (2016). Microstructure, defects and mechanical behavior of beta-type titanium porous structures manufactured by electron beam melting and selective laser melting. Acta Mater..

[12] Liu Y.J., Wang H.L., Li S.J., Wang S.G., Wang W.J., Hou W.T., Hao Y.L., Yang R., Zhang L.C. (2017). Compressive and fatigue behavior of beta-type titanium porous structures fabricated by electron beam melting. Acta Mater..

[13] Osetsky Y.N., Bacon D.J. (2010). Atomic-scale mechanisms of void hardening in bcc and fcc metals. Philos. Mag..

[14] Kumar K.S., Duesbery M.S., Louat N.P., Provenzano V., DiPietro M.S. (2001). Microporous fine-grained copper: Structure and properties. Philos. Mag. A.

[15] Xu W., Lui E.W., Pateras A., Qian M., Brandt M.J. (2017). In situ tailoring microstructure in additively manufactured Ti-6Al-4V for superior mechanical performance. Acta Mater..

[16] Gong H., Rafi K., Gu H., Ram G.J., Starr T., Stucker B. (2015). Influence of defects on mechanical properties of Ti-6Al-4 V components produced by selective laser melting and electron beam melting. Mater. Des..

[17] Agius D., Kyriakos K., Chris W. (2018). Elastoplastic response of as-built SLM and wrought Ti-6Al-4V under symmetric and asymmetric strain-controlled cyclic loading. Rapid Prototyp. J..

[18] Gupta A., Bennett C.J., Sun W. (2021). The role of defects and characterisation of tensile behaviour of EBM Additive manufactured Ti-6Al-4V: An experimental study at elevated temperature. Eng. Fail. Anal..

[19] Chern A.H., Nandwana P., McDaniels R., Dehoff R.R., Liaw P.K., Tryon R., Duty C.E. (2020). Build orientation, surface roughness, and scan path influence on the microstructure, mechanical properties, and flexural fatigue behavior of Ti-6Al-4V fabricated by electron beam melting. Mater. Sci. Eng. A.

[20] Lu J.X., Chang L., Wang J., Sang L.J., Wu S.K., Zhang Y.F. (2018). In-situ investigation of the anisotropic mechanical properties of laser direct metal deposition Ti-6Al-4V alloy. Mater. Sci. Eng. A.

[21] Yang Z.W., Fu L.Q., Wang S.L., Zhang M., Wang Y., Ma Z.Q., Wang D.P. (2021). Balance of strength and plasticity of additive manufactured Ti-6Al-4V alloy by forming TiB whiskers with cyclic gradient distribution. Addit. Manuf..

